# Coincidence of remission of postpartum Graves' disease and use of omega-3 fatty acid supplements

**DOI:** 10.1186/1756-6614-4-16

**Published:** 2011-11-16

**Authors:** Sarah J Breese McCoy

**Affiliations:** 1 OSU Center for Health Sciences and College of Osteopathic Medicine and Surgery, 1111 W. 17th St., Tulsa, Oklahoma 74107, USA

**Keywords:** Graves Disease, omega-3 fatty acids, postpartum thyroiditis, hyperthyroidism

## Abstract

I developed Graves' Disease four months postpartum. After one year on propylthiouracil, I learned that omega-3 fatty acids may reduce inflammation associated with certain autoimmune disorders, although no investigations for thyroiditis have been reported. Within eight weeks of beginning flaxseed oil supplements, TSH levels normalized, but fell somewhat when flaxseed was decreased and PTU discontinued. During another pregnancy, plasma TSH normalized, but was again suppressed by four weeks postpartum, then undetectable by four months. This time, flaxseed supplementation alone coincided with TSH normalization. Omega-3 fatty acids should be investigated as a potential treatment for autoimmune thyroid disease.

## Findings

I am a white female. In 2002 at age 38, I gave birth to my fifth child. About four months later, I developed heat intolerance, tremor, palpitations, increased frequency of bowel movements, and elevated heart rate. Plasma TSH was undetectable, and plasma T4 was elevated at 2.41 ng/dL (reference range 0.65-1.50 ng/dL). In addition, plasma thyrotropin binding inhibitory immunoglobulin level was elevated at 21.4% (reference range < 10%). Information on the lab results printout stated, "This test measures the percent inhibition of 125I-TSH binding to the thyroid TSH receptor. Graves Disease patients show an inhibition of 10-100%." My endocrinologist prescribed 10 mg methimazole twice per day, but a rash developed, so I was switched to propylthioruacil (PTU), 150 mg twice per day. (Thyroid ablation with radioactive iodine was not an option during breastfeeding.) After about eight weeks when plasma T4 levels came within normal range (1.1 ng/dL, reference range 0.7-1.9 ng/dL), I began to tapering off the PTU without the physician's consent, but T4 levels began rising, and TSH remained undetectable. I resumed taking PTU at 50-100 mg/day. At this dosage, TSH was still undetectable, but T4 remained within normal range, and I felt well. The endocrinologist would have preferred that I take enough PTU to bring TSH up to the normal range, but he continued to monitor me.

A thyroid uptake and scan were performed at about six months after beginning PTU therapy. The 24-hour uptake was elevated at 54.5 percent, and there was homogeneous distribution of the iodine radioisotope within a mildly enlarged gland, consistent with Graves' disease.

Approximately one year into the PTU treatment, I became aware that omega-3 fatty acids are thought to reduce the inflammation associated with certain autoimmune disorders, such as rheumatoid arthritis [1]. They have also been investigated with some success for such conditions as Crohn's disease, psoriasis, lupus erythematosus, and multiple sclerosis [2]. N-3 fatty acids are thought to exert their effects by suppressing the proliferation of T lymphocytes and autoantibodies [3]. Further, animal studies have shown that consumption of omega-3 alpha-linolenic acid leads to a change in the fatty acid composition of immune cell membranes in a dose dependent manner. As long-chain polyunsaturated omega-3 fatty acids (LCPUFA) are incorporated into cell membranes, less arachidonic acid is produced, and the inflammatory response is thus inhibited [4-8]. Unfortunately, no reports could be found regarding investigations of their use for autoimmune hyperthyroidism.

With this information in mind, I began a regimen of flaxseed oil supplements, 5-1,000 mg tablets twice a day. Flaxseed oil is over 50% omega-3 fatty acids (mainly alpha-linolenic acid), but it also contains about 15% omega-6 fatty acids (mainly linoleic acid) [9]. Within approximately eight weeks, TSH levels had normalized for the first time (0.311 uIU/mL, reference range 0.300-5.00 uIU/mL). PTU was then discontinued, and flaxseed oil tapered to less than half the original dose, but TSH levels slipped below normal again within six months (0.29 uIU/mL, reference range 0.35-5.00 uIU/mL). Symptoms were mild, and T4 was within normal range (1.0 ng/dL, reference range 0.7-1.9 ng/dL), so I declined to restart PTU therapy.

The following year, at about twenty weeks gestation during a sixth pregnancy, plasma levels of TSH normalized (0.726 uIU/mL, reference range 0.300-5.000 uIU/mL). This was not unexpected, because gestational increases in maternal plasma progesterone and estrogen concentrations typically lead to a suppression of the immune response, such as a shift in the population of T lymphocytes from Th1 to Th2 autoimmune responses. These changes lessen the probability of maternal rejection of the histoincompatible fetus and placenta. However, in the postpartum period, a shift back to a more robust and sensitive immune response favors occurrence or recurrence of postpartum thyroiditis [10,11]. Sure enough, by the fourth week postpartum, my TSH was again suppressed (0.174 uIU/mL, reference range 0.300-5.000 uIU/mL), becoming undetectable by four months postpartum. This time, however, plasma T4 remained within normal range (1.7 ng/dL, reference range 0.7-1.9 ng/dL). Although the physician advised me to take a low dose of PTU, I was experiencing no noticeable symptoms of hyperthyroidism and declined the prescription due to breastfeeding. As before, I then restarted a flaxseed regimen at about six months postpartum (this time, three tablespoons of whole seed on cereal each morning). My condition began improving, and plasma TSH normalized within six months (0.57 uIU/mL, reference range 0.35-5.00 uIU/mL). Flaxseed was then discontinued, and there has been no recurrence over the following four plus years.

Admittedly, about one third of patients using antithyroid agents can expect to experience a lasting remission [11]. However, in the second case, my remission occurred when I was not taking PTU, but not until beginning flaxseed therapy. Further, even during the first case, normalization of plasma TSH did not occur during the first year of PTU therapy alone, but was achieved within eight weeks of addition of a rich source of omega-3 fatty acids, flax seeds, to the PTU therapy.

There is insufficient evidence to conclude that my remission was caused by exposure to omega-3 fatty acids. However, the seemingly unlikely coincidences suggest a possible cause and effect relationship. The critical point is that a clinical trial of omega-3 fatty acid supplements for postpartum thyroiditis or Graves' disease seems warranted. Such a trial could be done while continuing other therapies such as methimazole or PTU, and would involve minimal risk at minimal expense. Clinical studies would be well advised to establish a secure dose of daily intake of omega-3 fatty acids, as well as the relative proportions of omega-3 and omega-6 fatty acids. Low doses of antioxidants, such as vitamin E or certain flavonoids, might also be added, because they may protect the body against the oxidative stress that omega-3 fatty acids can cause [12].

If a safe, easy, and cost effective new treatment could be found that increases a patient's odds of complete remission of Graves' disease, more people would be spared ablation therapy and lifelong thyroxine supplementation.

## Competing interests

The author declares that she has no competing interests.

## Author information

SJB McCoy is an adjunct professor of physiology at Oklahoma State University Center for Health Sciences in Tulsa. She has conducted clinical research and published a variety of papers in the field of reproductive endocrinology and postpartum depression.
